# IFNγ Production by Functionally Reprogrammed Tregs Promotes Antitumor Efficacy of OX40/CD137 Bispecific Agonist Therapy

**DOI:** 10.1158/2767-9764.CRC-23-0500

**Published:** 2024-08-12

**Authors:** Charlotte J. Imianowski, Paula Kuo, Sarah K. Whiteside, Teresa von Linde, Alexander J. Wesolowski, Alberto G. Conti, Alexander C. Evans, Tarrion Baird, Benjamin I. Morris, Nicole E. Fletcher, Jie Yang, Edmund Poon, Matthew A. Lakins, Masahiro Yamamoto, Neil Brewis, Michelle Morrow, Rahul Roychoudhuri

**Affiliations:** 1 Department of Pathology, University of Cambridge, Cambridge, United Kingdom.; 2 Immunology Programme, Babraham Institute, Babraham Research Campus, Cambridgeshire, United Kingdom.; 3 F-Star Therapeutics, Babraham Research Campus, Cambridgeshire, United Kingdom.; 4 Department of Immunoparasitology, Research Institute for Microbial Diseases, Osaka University, Osaka, Japan.; 5 Laboratory of Immunoparasitology, WPI Immunology Frontier Research Center, Osaka University, Osaka, Japan.; 6 Department of Immunoparasitology, Center for Infectious Disease Education and Research, Osaka University, Osaka, Japan.; 7 invoX Pharma, Cambridge, United Kingdom.

## Abstract

Regulatory T cells (Treg) are highly enriched within many tumors and suppress immune responses to cancer. There is intense interest in reprogramming Tregs to contribute to antitumor immunity. OX40 and CD137 are expressed highly on Tregs, activated and memory T cells, and NK cells. In this study, using a novel bispecific antibody targeting mouse OX40 and CD137 (FS120m), we show that OX40/CD137 bispecific agonism induces potent antitumor immunity partially dependent upon IFNγ production by functionally reprogrammed Tregs. Treatment of tumor-bearing animals with OX40/CD137 bispecific agonists reprograms Tregs into both fragile Foxp3^+^ IFNγ^+^ Tregs with decreased suppressive function and lineage-instable Foxp3^−^ IFNγ^+^ ex-Tregs. Treg fragility is partially driven by IFNγ signaling, whereas Treg instability is associated with reduced IL2 responsiveness upon treatment with OX40/CD137 bispecific agonists. Importantly, conditional deletion of *Ifng* in Foxp3^+^ Tregs and their progeny partially reverses the antitumor efficacy of OX40/CD137 bispecific agonist therapy, revealing that reprogramming of Tregs into IFNγ-producing cells contributes to the anti-tumor efficacy of OX40/CD137 bispecific agonists. These findings provide insights into mechanisms by which bispecific agonist therapies targeting costimulatory receptors highly expressed by Tregs potentiate antitumor immunity in mouse models.

**Significance:**

The bispecific antibody FS120, an immunotherapy currently being tested in the clinic, partially functions by inducing anti-tumor activity of Tregs, which results in tumor rejection.

## Introduction

Immunomodulatory therapies targeting the PD1/PDL1 and CTLA4 effector T (Teff)–cell inhibitory signaling pathways induce striking objective clinical responses in certain cancer types but are ineffective at inducing durable responses in a majority of patients (1). These findings provide a rationale for developing new mechanistically distinct immunomodulatory therapies for cancer. CD4^+^ regulatory T cells (Treg), in which development is dependent on the transcription factor Foxp3, powerfully suppress Teff cells and prevent immune-mediated rejection of tumors (2, 3). Low Treg-to-Teff cell ratios are associated with favorable prognosis and survival in the absence of immunotherapeutic treatment in ovarian cancer (4, 5), breast cancer (6), non–small cell lung carcinoma (7), hepatocellular carcinoma (8), renal cell carcinoma (9), pancreatic cancer (10), gastric cancer (11), cervical cancer (12), and colorectal carcinoma (13). In murine tumor models, ablation of Tregs results in activation of CD4^+^ or CD8^+^ Teff cells and rejection of solid tumors (14–17). Tregs therefore represent an attractive target for immunotherapy. Despite recent advances in developing an optimized anti-CD25 antibody with the potential to selectively deplete Tregs *in vivo* (18), there are currently no Treg-targeting therapies approved for clinical use.

The immunosuppressive tumor microenvironment, to which Tregs are a major contributing factor, presents a significant barrier to effective antitumor immunity (19). The stability of Tregs defined by their ability to maintain expression of Foxp3, is a requirement to sustain their suppressive function (20). Tregs exhibit high levels of lineage stability under steady state conditions and in experimental models of Th1 and autoimmune inflammation (21). However, Treg lineage instability has been observed in some conditions. Zhou and colleagues (22) observed accumulation of “Ex-Foxp3” IL17-expressing cells in inflamed joints in response to synovial IL6. Conversion of purified populations of Foxp3^+^ Tregs into Foxp3^−^ cells has also been observed in adoptive transfer models, both during conditions of extreme inflammation induced through allogeneic bone marrow transplantation (23) and extensive lymphopenia-induced proliferation (24).

Tregs can also lose suppressive capacity or gain the ability to produce effector cytokines while retaining Foxp3 expression (20). This has been referred to as Treg fragility. Agonists of the costimulatory receptor Glucocorticoid-Induced TNFR-Related (GITR) have been proposed to drive fragility of Tregs through the reduction of Helios expression and induction of IFNγ and TNFα expressions by Foxp3^+^ Tregs (25). Neuropilin 1, a receptor expressed by Tregs, suppresses Akt signaling at inflammatory sites to promote Treg functional stability (26). This is particularly the case in the context of tumor-associated Tregs, such that the loss of neuropilin 1 drives Treg fragility and promotes antitumor responses (27).

CD137 (4-1BB), a member of the TNF receptor (TNFR) superfamily, is expressed on several cell types, including activated T cells and Tregs. CD137 costimulates activated T cells, resulting in proliferation, memory cell formation, increased survival, and the production of proinflammatory cytokines (28). CD137 agonistic antibodies can provoke rejection of tumors in multiple mouse models but nonspecific agonism results in generalized T cell activation, cytokine release, and systemic inflammation (29). Despite the initial signs of efficacy, clinical development of the CD137 agonist urelumab has been hampered by inflammatory liver toxicity at moderate systemic doses, whereas utomilumab functions as a less potent agonist despite a favorable safety profile (30). Thus, there is a need to develop more specific approaches to activate CD137 signaling.

OX40 is also a costimulatory molecule belonging to the TNFR superfamily (31). It is constitutively expressed by Tregs but not by resting conventional T (Tconv) cells (32). Upon immune activation, however, activated Teff cells gain OX40 expression and costimulatory signaling via OX40/OX40 ligand engagement, which supports survival, differentiation, and memory phenotype transition (33). Stimulation of OX40 on Tregs has been shown to interfere with their regulatory functions (34–36), and OX40 signaling may contribute to competitive fitness in cellular reconstitution models, indicating an important role in Treg proliferation and survival (37). Tregs expanded by OX40 stimulation are poorly suppressive, due to a relative deficiency of IL2 signaling and an “exhausted” phenotype, which requires exogenous IL2 to overcome (38). Clinical trials using OX40 agonist antibodies exhibited peripheral CD4^+^ and CD8^+^ T-cell activation and proliferation without toxicity but showed limited clinical efficacy (39, 40). Mechanisms to improve the clinical efficacy of agonist antibodies are therefore of significant interest.

TNFR superfamily agonist antibodies generally require secondary cross-linking of antibody–receptor complexes to induce sufficient receptor clustering and activation by imitating the TNF superfamily ligand superclusters (41). Secondary cross-linking *in vivo* usually requires interaction of agonistic antibodies with Fcγ receptors (FcγR; ref. 42). The low-affinity interaction between FcγRs and the fragment crystallizable (Fc) region of IgG antibodies and the relatively low abundance of FcγR-expressing cells in the tumor microenvironment are both limitations to agonist activity and resulting antitumor immunity. Consideration should also be given to the antibody-mediated effector functions that are induced by interaction with FcγRs which could result in the depletion of tumor-reactive T cells (43). Methods to improve the clinical activity of agonist antibodies targeting receptors like OX40 and CD137 without inducing toxicity are therefore required.

Targeting both OX40 and CD137 together is of interest due to their overlapping but distinct expression profiles (44). FS120 is a novel tetravalent bispecific antibody targeting OX40 and CD137 (45). Previous work has shown that it can activate both CD4^+^ and CD8^+^ T cells. The introduction of FcγR-disabling LALA mutations (46) prevents antibody cross-linking through FcγR binding and limits receptor cross-linking until co-engagement of the bispecific antibody with both OX40 and CD137, most frequently when they are coexpressed on the same cell. This provides cell-type specificity and avoids the depletion of OX40- or CD137-expressing cells. Importantly, a mouse-specific surrogate version of FS120 (hereinafter referred to as FS120m) induced antitumor activity in a CT26 tumor model, which was associated with reduced liver T-cell infiltration when compared with other agonist antibodies targeting CD137, as an indication of reduced off-target toxicity (45).

Here, we show that the efficacy of OX40/CD137 bispecific agonist FS120m is partially dependent upon Treg functional reprogramming into fragile and lineage-instable Tregs producing IFNγ. Using *Foxp3* fate-tracking reporter mice, we find that Tregs from mice treated with FS120m undergo reprogramming into fragile Foxp3^+^ IFNγ^+^ cells with decreased suppressive function and instable IFNγ^+^ Foxp3^−^ exTregs. Treg fragility is partially dependent upon IFNγ signaling, whereas Treg lineage instability is associated with reduced CD25 expression by Tregs upon FS120m treatment *in vivo*. The efficacy of FS120m therapy is abolished upon antibody blockade of IFNγ, and strikingly, the conditional deletion of *Ifng* in Foxp3^+^ Tregs and their progeny in large part reverses the antitumor efficacy of OX40/CD137 bispecific agonist FS120m.

## Materials and Methods

### Mice and reagents

*Foxp3* ^EGFP-Cre-ERT2^, *Rosa26*^flSTOPfl-tdRFP^, *Foxp3*^IRES-EGFP^, *Ptprc*^a^ (CD45.1), and *Rag2*^−/−^ mice were obtained from The Jackson Laboratory. *Ifng*^flox/flox^ mice were generated as previously described (47). C57BL/6 mice were purchased from Charles River Laboratories and housed in the University Biological Services (UBS) Gurdon facility (University of Cambridge) for at least 1 week of acclimatization period prior to the start of the experiment. Genetically modified animals were crossed in the Biological Support Unit at the Babraham Institute and at the UBS Gurdon facility to obtain *Ifng*^WT^*Foxp3*^EGFP-Cre-ERT2^*Rosa26*^flSTOPfl-tdRFP^ and *Ifng*^flox/flox^*Foxp3*^EGFP-Cre-ERT2^ mice, respectively. Littermate controls or age- and sex-matched animals were used in experiments as indicated. All mice were housed at the UBS Gurdon facility or the Babraham Institute Biological Support Unit. All animal experiments were conducted in accordance with UK Home Office guidelines and were approved by the Babraham Institute and/or The Animal Welfare and Ethics Review Board, University of Cambridge. The mice were genotyped by TransnetYX (Memphis, TN). For induction of Cre-ERT2–mediated recombination, the mice either received three doses of tamoxifen (13258, the Cayman Chemical Company) by oral gavage on the first, second, and fourth day of the experiment or were fed tamoxifen-containing food (TD130858, Envigo). To mitigate neophobic effects, mice were given non–tamoxifen-containing food mashed with Nesquik strawberry milkshake mix (Nestlé) for 10 days prior to initiation of treatment with tamoxifen-containing food mashed with Nesquik strawberry milkshake mix. Tamoxifen by oral gavage was administered in 8-mg doses dissolved in 200 μL corn oil (C8267, Sigma-Aldrich).

### Tumor induction

MC38 colorectal carcinoma cells were purchased from Kerafast in 2018 and authenticated as *Mycoplasma*-free using PCR testing on June 25 of the same year. After thawing, the cells were passaged no more than twice in DMEM (Invitrogen, Thermo Fisher Scientific) supplemented with 10% FBS and antibiotics prior to their use in experiments. Mice were injected subcutaneously with 3 × 10^5^ cells under isofluorane anesthesia. FS120m (1 mg/kg), anti-IFNγ (10 mg/kg, clone XMG1.2, Bio X cell), and/or anti-PD1 (10 mg/kg, clone RMP1-14, Bio X cell) or their respective isotype controls were injected intraperitoneally. FS120m was dosed on days 10, 12, and 14 after tumor implantation, and anti-IFNγ or anti-PD1 was given on days 10, 14, 18, and 21 after tumor implantation. rIL2 (1 μg, PeproTech, 212-12) and anti-IL2 (5 μg, clone JES6-1, Thermo Fisher Scientific) complex was given via intraperitoneal injection on days 17, 18, and 19 after tumor injection. The tumor volume was calculated as length × width^2^.

### Antibodies

The bispecific antibody targeting mouse OX40 and CD137 (FS120m) and its isotype control were designed, produced, subjected to quality control, and provided by F-Star Therapeutics (45). All antibodies were diluted from their stock concentrations in PBS and injected in volumes of 100 μL at the timepoints indicated above.

### Lymphocyte isolation

Single-cell suspensions from lymphoid tissues were prepared by mechanical dissociation through 40-μm cell strainers (Thermo Fisher Scientific). Erythrocytes in splenocyte and blood samples were lysed using ice-cold ACK lysing buffer (Gibco) for 3 to 5 minutes before the samples were filtered for a second time. Tumors were dissected and minced in media containing 20 μg/mL DNase I (Roche) and 1 mg/mL collagenase (Sigma-Aldrich), followed by incubation with agitation for 30 minutes at 37°C. Digested tissue was then passed through a 40-μm cell strainer and pelleted by centrifugation. Lymphocytes were further isolated by using a density gradient with Percoll solution (Cytiva, 17-0891-01). Nine parts of Percoll were mixed with one part 10× PBS to create a stock solution. The pelleted cells were resuspended with 5 mL of 40% Percoll (made by mixing 40% stock solution with 60% complete RPMI 1640; Invitrogen, Thermo Fisher Scientific), an then a 1 mL underlayer of 80% Percoll was gently introduced. Centrifugation was carried out for 23 minutes at 2,300 rpm at room temperature. Lymphocytes were isolated from the interface and washed with PBS.

### Flow cytometry

Cells requiring intracellular staining of cytokines were stimulated prior to flow cytometry analysis using phorbol 12-myristate 13-acetate, ionomycin, and brefeldin A (all from Sigma-Aldrich) for 4 hours in RPMI supplemented with 10% FBS and antibiotics. Viable cells were discriminated by staining with Fixable Viability Dye eFluor 780 (eBioscience) alongside surface-only antibodies in PBS containing 2% FBS and 0.2 mmol/L EDTA (Invitrogen). Some viable cells were discriminated by staining with Live/Dead Blue or Live/Dead Violet fixable stain in PBS (Thermo Fisher Scientific), or with DAPI. Samples for which the preservation of fluorescent proteins was not necessary were then fixed using the eBioscience Foxp3/Transcription Factor Staining Buffer Set (Invitrogen, Thermo Fisher Scientific) before the rest of the markers were stained intracellularly overnight (48). For samples that required preservation of fluorescent proteins during intracellular staining, the cells were fixed using the BD Fixation/Permeabilization Solution Kit (BD Biosciences). For this, after the first fixation step, the cells were frozen overnight before thawing to permeabilize, refixing, and then staining intracellularly. Samples were analyzed using the BD LSRFortessa (BD Biosciences), the Beckman CytoFLEX (Beckman Coulter), the BD LSR II (BD Biosciences), or Cytek Aurora spectrum analyzer (Cytek Biosciences). Data and representative flow plots were analyzed and generated using FlowJo software (TreeStar LLC). Graph generation and statistical analysis were conducted using GraphPad Prism.

Antibodies used for flow cytometry are listed as follows: anti–CD45.1 APC (A20), anti–CD45.2 eFluor 506 (104), anti–CD62L eFluor 450 (MEL-14), anti–OX40 BV711 (OX86), anti–CD44 PerCp-Cy5.5 (IM7), anti–CD137 PE (17B5), anti–CD4 eFluor 450, PE-Cy7 or PerCp-Cy5.5 (RM4-5), anti–CD8a APC-eFluor 780 or APC (53-6.7), anti–CD25 PE-Cy7 (PC61.5), anti–Foxp3 FITC or APC (FJK-16s), anti–IFNγ PerCp-Cy5.5 or APC (XMG1.2), and anti–TNFα PE-Cy7 (TN3-19) from eBioscience; anti–CD62L PerCp-Cy5.5 (MEL-14), anti–CD3e BV785 (145-2C11), anti–CD4, BV650, or Alexa Fluor 700 (RM4-5), anti–CD4 Alexa Fluor 594 or PE-Cy7 (GK1.5), anti–CD8a BV650 (53-6.7), anti–TCRβ PerCp-Cy5.5, PE or PE-Cy7 (H57-597), anti–IFNγ FITC (XMG1.2), and anti–IL2 BV421 (JES6-5H4) from BioLegend; and anti–CD3e BV650 (145-2C11), anti–CD44 BV786 (IM7), and anti–CD4 BUV395 (GK1.5) from BD Biosciences.

### FACS

Pre-enrichment of CD4^+^ T cells from single-cell suspensions was done using the MagniSort Mouse CD4^+^ T Cell Enrichment Kit (Invitrogen, Thermo Fisher Scientific) according to the manufacturer’s protocol. Any markers required for cell sorting were stained using flow cytometry cell surface antibodies after enrichment and incubated alongside eFluor 780 fixable viability dye for discrimination of dead cells. Cell sorting was performed using a BD Aria instrument (BD Biosciences). The cells were sorted into solutions of complete RPMI 1640 supplemented with an additional 25% FBS (Sigma-Aldrich) before being prepared for experiments as described below.

### Treg suppression assay

The suppressive capacity of Tregs treated with FS120m was tested as previously described (49). *Foxp3*^IRES-EGFP^ mice were dosed with FS120m or isotype control antibody (1 mg/kg) intraperitoneally on days 1, 3, and 5. On day 8, splenic GFP^+^ Tregs were sorted by FACS from *Foxp3*^IRES-EGFP^ mice and naïve CD4^+^ Tconv cells (CD25^−^ CD44^−^ CD62L^+^) were sorted from wild-type (WT) CD45.1 mice. The sorted naïve CD4^+^ Tconv cells were stained with CellTrace Violet according to the manufacturer’s protocol (Thermo Fisher Scientific). Tregs and Tconv cells were plated in a 1:8 ratio (1.25 × 10^4^ Tregs with 1 × 10^5^ Tconv cells) in the presence of anti-CD3 (BioLegend, 1 μg/mL) and 5 × 10^4^*Rag2*^−/−^ antigen-presenting cells. Naïve Tconv cells cultured without Tregs were used as the proliferating control. Cell division was evaluated by flow cytometry after 4 days of culture.

### IL2 *in vitro* culture assay

*Foxp3* ^IRES-GFP^ mice were dosed with FS120m or isotype control antibody (1 mg/kg) intraperitoneally on days 1, 3, and 5, and on day 9, GFP^+^ Tregs were sorted by FACS. The sorted Tregs were then cultured in complete RPMI supplemented with IL2 in indicated concentrations for 72 hours before analysis by flow cytometry.

### DNA isolation and PCR genotyping

*Ifng* ^fl/fl^ *Foxp3* ^EGFP-Cre-ERT2^ and *Ifng*^WT^*Foxp3*^EGFP-Cre-ERT2^ mice were treated with tamoxifen by oral gavage on days 1, 2, and 4 using 8 mg doses dissolved in 200 μL corn oil (C8267, Sigma-Aldrich). The spleens were isolated on day 11 and stained directly for FACS after erythrocyte lysis. CD8^+^ T cells, CD4^+^ Foxp3-GFP^−^ Tconv cells, and CD4^+^ Foxp3-GFP^+^ Tregs were sorted on a BD Aria instrument (BD Biosciences). DNA was isolated from these populations using the DNeasy Blood & Tissue Kit (QIAGEN) according to the manufacturer’s protocol. PCR mixes were 20 μL in total volume and contained the following: 20 ng template DNA, 200 μmol/L dNTPs, 0.5 μmol/L primers, 4 μL 5× Phusion high-fidelity buffer, 3% DMSO, and 0.4 μL Phusion Polymerase (Thermo Fisher Scientific). All PCRs were run on a Bio-Rad T100 Thermal Cycler using the following primers: *Ifng*^flox^-F: 5′-ATC​AAG​CTG​CCT​CCC​GTA​TGT​GTT​T-3′ and *Ifng*^flox^-R: 5′-TGA​GTC​ATC​TGT​AGT​CAG​CGT​TCC​T-3′. PCR thermal cycling was set as follows: initial denaturation at 98°C for 30 seconds, followed by 30 cycles of 98°C for 10 seconds, 68°C for 30 seconds, and 72°C for 30 seconds/kb, and then a final extension of 72°C for 5 minutes. Amplicons were visualized by agarose gel electrophoresis.

### Statistical testing

Data were analyzed using unpaired two-tailed Student *t* test, one-way or two-way ANOVA as indicated. Bonferroni correction or Tukey correction for multiple comparisons were applied where stated. For tumor experiments, female mice were randomized, and the operator was blinded to genotype while conducting tumor measurements. Mice were randomised to treatment groups following measurement on days 7 to 9, and animals without tumors were removed from the experiment before treatment started on day 10. Samples used for flow cytometry were also blinded to genotype and treatment group during dissection and remained so until the final stage of analysis. Experimental sample sizes were chosen using power calculations or preliminary experiments, or were based on previous experience of variability in similar experiments. Samples which had undergone technical failure during processing were excluded from subsequent analysis.

### Data availability

The data generated in this study are available within the article and its supplementary data files.

## Results

### OX40 and CD137 are highly coexpressed on tumor-associated Tregs

**Figure 1:**
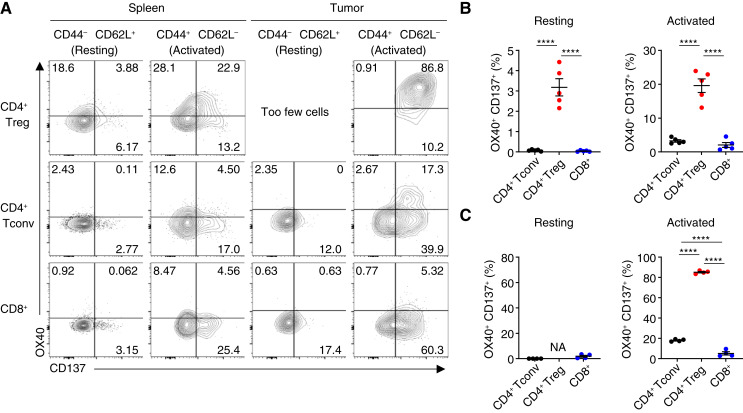
OX40 and CD137 are highly coexpressed on tumor-associated Tregs. **A,** Representative plots showing expression of OX40 and CD137 on resting and activated CD4^+^ Tregs, CD4^+^ Tconv cells, and CD8^+^ T cells from the spleens and tumors of mice inoculated with MC38 cells. **B** and **C,** Quantification of resting CD44^−^ CD62L^+^ (left) and activated CD44^+^ CD62L^−^ (right) cells within the indicated T-cell populations in the spleen (**B**) and tumor (**C**). Data in **B** and **C** were analyzed by one-way ANOVA with Tukey correction for multiple comparisons. Bars and error are mean and SEM. ****, *P* ≤ 0.0001.

We first examined the expression profile of OX40 and CD137 on different T-cell subsets within tumors and lymphatics, given that FS120 activity is enriched on cells coexpressing OX40 and CD137. We injected MC38 colorectal carcinoma cells subcutaneously into C57BL/6 mice and allowed tumors to develop for 18 days. Resting (CD44^−^ CD62L^+^) and activated (CD44^+^ CD62L^−^) CD8^+^ T cells, CD4^+^ Tconv cells, and Tregs were analyzed by flow cytometry (Fig. 1A). We found that Tregs had the highest percentage of OX40^+^ CD137^+^ double-positive cells in both spleens and tumors, whereas the percentage of OX40^+^ CD137^+^ cells increased among activated compared with resting CD8^+^ and CD4^+^ Tconv cells (Fig. 1B and C). Strikingly, more than 80% of activated Tregs from tumors were double-positive for OX40 and CD137 expression. Tregs also tended to have the largest percentage of OX40^+^ and CD137^+^ single-positive cells among the cell types examined (Supplementary Fig. S1). This led us to hypothesize that therapies targeting coexpression of these costimulatory molecules may have a greater effect on Tregs than they do on CD4^+^ Tconv and CD8^+^ T cells.

### OX40/CD137 dual agonism with FS120m drives functional fragility and lineage instability of Tregs

Tregs are a remarkably stable lineage, capable of maintaining expression of Foxp3 even in the presence of inflammatory signaling (21). However, in certain settings, a proportion of Tregs, known as exTregs, lose Foxp3 expression and acquire a proinflammatory phenotype (20, 26). To assess lineage stability of Tregs when treated with OX40/CD137 bispecific agonist FS120m, we utilized *Foxp3*^EGFP-Cre-ERT2^*Rosa26*^fl-STOP-fl-tdRFP^ lineage-tracking reporter mice (50). In these mice, Cre recombinase activity induced within *Foxp3*^+^ Treg cells upon tamoxifen treatment removes a stop codon flanked by LoxP sites in the *Rosa26* locus to induce red fluorescent protein (RFP) expression, which is maintained even if *Foxp3* is downregulated. Therefore, after tamoxifen-induced labeling, Tregs become GFP^+^ and RFP^+^ but Tregs which subsequently lose *Foxp3* expression (known as exTregs) become RFP^+ and GFP^−^,^ indicating lineage instability.

**Figure 2:**
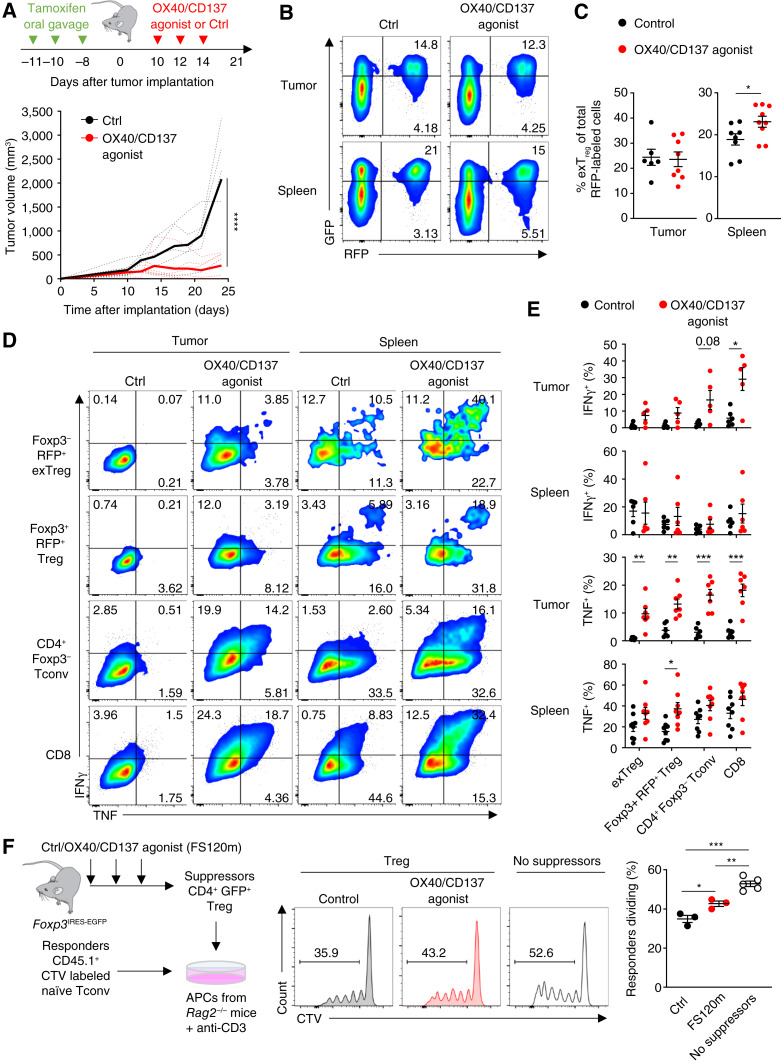
OX40/CD137 bispecific agonist (FS120m) drives functional fragility and lineage instability of Tregs. **A,** Schema (top) showing tamoxifen and FS120m treatment schedule. Tumor measurements (bottom) at indicated timepoints after MC38 tumor implantation. (Solid line, mean values; dotted lines, individual mouse tumor curves.) **B,** Representative plots showing percentages of CD4^+^ GFP^+^ RFP^+^ Tregs and CD4^+^ RFP^+^ GFP^−^ exTregs of total CD4^+^ T cells in the spleens and MC38 tumors of *Foxp3*^EGFP-Cre-ERT2^*Rosa*^flSTOPfl-RFP^ reporter mice after treatment. **C,** Quantification of the percentage of RFP^+^ single-positive exTregs (GFP^−^) out of total RFP^+^ cells in spleens and tumors. **D,** Representative plots showing the production of IFNγ and TNF by indicated cell subsets from the spleens and tumors of reporter mice. **E,** Quantification of IFNγ and TNF production by indicated cell types from the spleens and tumors of MC38 tumor–bearing mice at day 21 after tumor implantation. **F,** Schema showing setup of Treg suppression assay (left), representative flow plots (center), and replicate measurements (right) showing percentage of dividing responders in indicated conditions. Data were analyzed by two-way ANOVA with Šídák correction for multiple comparisons (**A**), unpaired Student *t* test (**C**), with Bonferroni–Dunn correction for multiple comparisons (**E**), and ordinary one-way ANOVA with Tukey correction for multiple comparisons (**F**). Bars and error are mean and SEM. *, *P* ≤ 0.05; **, *P* ≤ 0.01; ***, *P* ≤ 0.001; ****, *P* ≤ 0.0001. Ctrl, control.

We treated *Foxp3*^EGFP-Cre-ERT2^*Rosa26*^flSTOPfl-tdRFP^ mice with tamoxifen 10 days prior to injection with MC38 cells. The tumor-bearing mice were then treated with FS120m or isotype control antibodies from day 10 after tumor injection. Strikingly, treatment with FS120m resulted in significantly reduced tumor growth using the MC38 tumor model, similar to previous findings using a CT26 tumor model (Fig. 2A; ref. 45). At 21 days after tumor injection, Tregs from both the tumor and spleen were assessed for GFP and RFP expression (Fig. 2B). We found that the percentage of exTregs (defined as the percentage of RFP^+^ GFP^−^ cells out of all RFP^+^ cells) was significantly increased in the spleens of mice treated with FS120m, whereas in tumors, there was a trend toward an increase (Fig. 2C). The percentage of CD8^+^ T cells infiltrating tumors of FS120m-treated animals was also increased, but the percentage of Foxp3^+^ RFP^+^ Tregs was not significantly altered (Supplementary Fig. S2A and-S2B). Quantitation of the absolute numbers of *Foxp3*^−^ RFP^+^ exTreg and *Foxp3*^+^ RFP^+^ Treg populations revealed a significant increase in both cell types per gram of tumor in FS120m-treated mice, reflecting a general increase in lymphocyte infiltration induced by FS120m treatment. We also found that there was a significant increase in exTreg numbers in spleens of treated animals, but this was not accompanied by changes in the Foxp3^+^ RFP^+^ Treg population (Supplementary Fig. S2C). These findings indicate that treatment with FS120m induces instability of Tregs, associated with an increase in the abundance of exTregs, but this is not associated with a concomitant decrease in Foxp3^+^ Tregs.

The increase in Foxp3^−^ exTregs indicated that FS120m treatment can induce Treg lineage instability, which has been associated with the upregulation of proinflammatory cytokine expression. We therefore assessed the expression of cytokines IFNγ and TNF in exTregs, Foxp3^+^ RFP^+^ Tregs, CD4^+^ Tconv cells and CD8^+^ T cells. FS120m treatment resulted in a general increase in proinflammatory cytokine expression in all cell types analyzed, particularly those within tumors (Fig. 2D and E). In particular, we noted an increase in the percentage of Foxp3^+^ RFP^+^ Tregs, alongside Foxp3^−^ exTregs, expressing these cytokines. This indicates that bispecific agonism of OX40 and CD137 with FS120m can induce Treg fragility, whereby Tregs expressing Foxp3 begin to express proinflammatory cytokines, in addition to inducing inflammatory cytokine expression by lineage-instable exTregs.

Importantly, we also noted that although there were general increases in the absolute numbers of cytokine-producing exTregs and Foxp3^+^ RFP^+^ Tregs in both tumor and the spleen, it was the “fragile” cytokine-producing Foxp3^+^ Tregs which were more numerous (Supplementary Fig. S2D and S2E).

To test the suppressive function of Tregs from mice receiving OX40/CD137 bispecific agonist treatment, we compared Tregs sorted from mice that received FS120m with those from control-treated animals for their ability to suppress the proliferation of naïve CD45.1 Tconv cells (49, 51). Consistent with evidence that Tregs from mice treated with FS120m are functionally fragile, we found that *Foxp3*^GFP+^ cells sorted from the spleens of *Foxp3*^IRES-EGFP^ mice that were given FS120m showed reduced suppressive capacity (Fig. 2F). Therefore, we hypothesized that FS120m acts via Tregs to promote antitumor immunity through a direct mechanism involving their production of proinflammatory cytokines and through an indirect mechanism which results from their impaired ability to suppress tumor-targeting Tconv cells.

### OX40/CD137 dual agonism results in decreased CD25 expression and diminished IL2 responsiveness of Tregs

**Figure 3:**
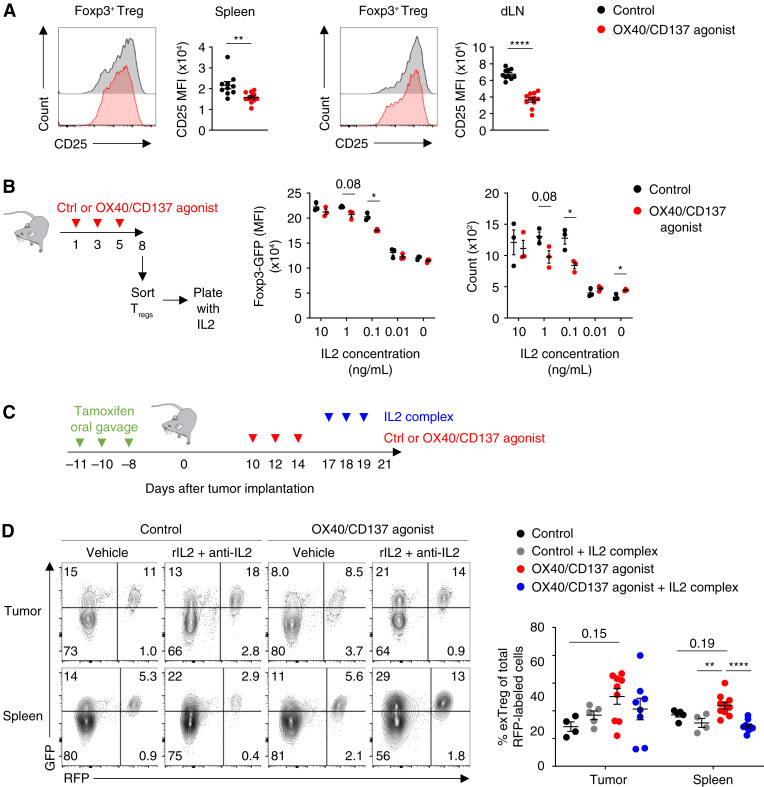
OX40/CD137 dual agonism results in decreased CD25 expression and IL2 responsiveness of Tregs. **A,** Representative histograms and replicate measurements showing CD25 expression on CD4^+^ Tregs from the spleen (left) and tumor-draining lymph node (right). **B,** Schema showing setup of *ex vivo* IL2 assay (left), replicate measurements of Foxp3-GFP expression (center), and Treg count (right) after 4 days in culture with the indicated concentrations of IL2. **C,** Schema representing treatment schedule of tamoxifen, FS120m, and rIL2/anti-IL2 mAb complex. **D,** Representative plots (left) and replicate measurements (right) of the percentage of RFP^+^ SP exTregs (GFP^−^) out of total RFP^+^ cells in the tumors and spleens of reporter mice treated according to **C**. Data were analyzed by unpaired Student *t* test (**A**), unpaired Student *t* test with Bonferroni–Dunn correction for multiple comparisons (**B**), and one-way ANOVA with Tukey correction for multiple comparisons (**D**). Bars and error are mean and SEM. *, *P* ≤ 0.05; **, *P* ≤ 0.01; ***, *P* ≤ 0.0001; ****, *P* ≤ 0.0001. Ctrl, control; dLN, tumor-draining lymph node.

We wished to investigate the mechanisms by which treatment with OX40/CD137 bispecific agonist FS120m induces Treg fragility and instability. It has been previously reported that blockade of IL2 signaling can result in Treg lineage instability (21). However, when we analyzed IL2 production by CD8^+^ T cells and CD4^+^ Tconv cells, we found that FS120m induced marginally increased expression among cells from the spleen and tumor-draining lymph nodes (Supplementary Fig. S3). CD25 is the high-affinity IL2 receptor expressed by most Tregs (52–54). We observed a significant reduction in CD25 expression on Tregs from FS120m-treated mice (Fig. 3A). We hypothesized that this could be responsible for the instable phenotype of Tregs induced by FS120m treatment and that treatment with excess exogenous IL2 would be able to overcome the deficiency in signaling and restabilize the cells.

First, we cultured GFP^+^ Tregs sorted from FS120m and control-treated *Foxp3*^IRES-EGFP^ animals *in vitro* in the presence of titrated quantities of IL2. The lowest concentrations of IL2 resulted in impaired survival of Treg cells, consistent with a critical role for IL2 in limiting Treg apoptosis (32). Tregs cultured with the highest concentrations of IL2 had improved survival and minimal evidence of lineage instability as indicated by high levels of Foxp3-GFP expression. Intriguingly, at intermediate concentrations of IL2, Tregs that had been sorted from mice treated with FS120m exhibited lower Foxp3-GFP expression and reduced cell counts than Tregs from control-treated animals (Fig. 3B). This indicated that excess IL2 can overcome lineage instability, associated with reduced CD25 expression, of Treg cells from FS120m-treated animals.

To test if excess IL2 administered *in vivo* is capable of reversing the instability phenotype of Tregs from FS120m-treated mice, we treated tumor-bearing *Foxp3*^EGFP-Cre-ERT2^*Rosa23*^flSTOPfl-tdRFP^ mice with FS120m or isotype control, then treated half of each group with a stabilised IL2:anti-IL-2 complex (rIL2 combined with an anti-IL2 mAb to increase half-life *in vivo*; ref. 55) on the last 3 days before analysis (Fig. 3C). We found that treatment of mice with FS120m and rIL2/anti-IL2 complex reversed the lineage instability of Tregs seen in FS120m-treated animals (Fig. 3D). This supports the hypothesis that FS120m acts to destabilize Tregs by decreasing IL2 signaling through reduction of surface expression of CD25, and this can be overcome by providing exogenous IL2, which increases signaling through the remaining surface receptors.

### OX40/CD137 dual-agonist–driven antitumor immunity is dependent upon IFNγ signaling

**Figure 4:**
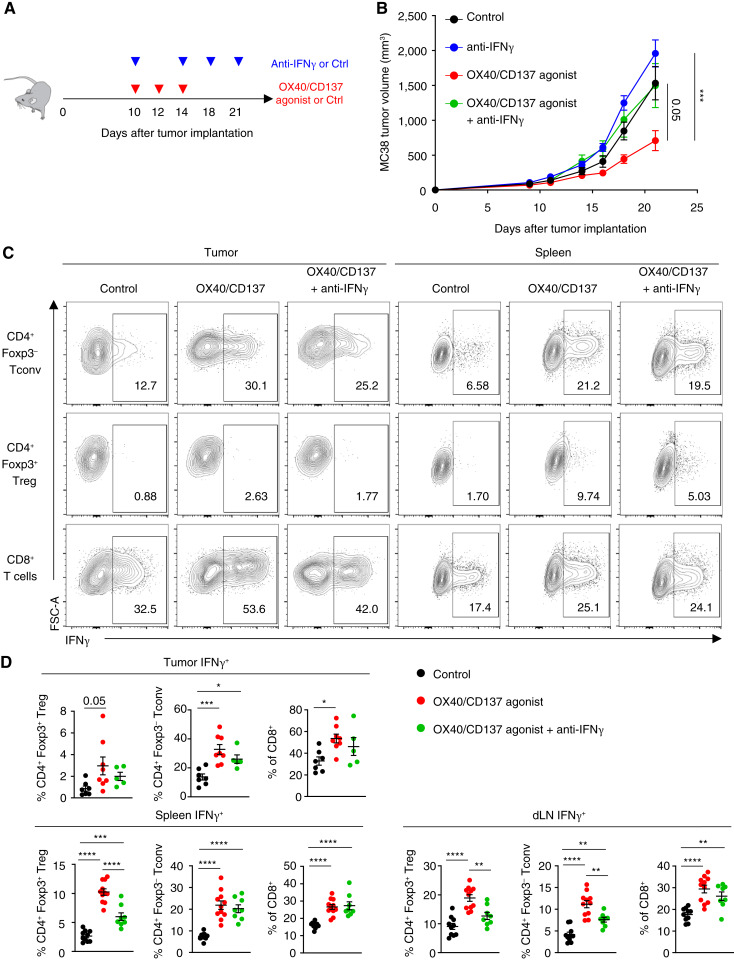
The antitumor efficacy of OX40/CD137 dual agonism is dependent upon IFNγ signaling. **A,** Schema representing treatment schedule of FS120m and anti-IFNγ. **B,** Tumor measurements at indicated timepoints after MC38 tumor implantation of the mice described in **A**. **C,** Representative plots from the tumor (left) and spleen (right) showing IFNγ^+^ cells in CD8^+^ T cells and indicated CD4^+^ T-cell populations from mice receiving the specified treatment. **D,** Replicate values of IFNγ^+^ cells in CD8^+^ T cells and the indicated CD4^+^ T-cell populations from the tumor, spleen, and tumor-draining lymph node of mice receiving the specified treatment. Data were analyzed by two-way ANOVA with Tukey correction for multiple comparisons (**B**) and ordinary one-way ANOVA with Tukey correction for multiple comparisons (**D**). Bars and error are mean and SEM. *, *P* ≤ 0.05; **, *P* ≤ 0.01; ***, *P* ≤ 0.001; ****, *P* ≤ 0.0001. Ctrl, control; dLN, tumor-draining lymph node; MFI, mean fluorescence intensity.

We observed that FS120m treatment induces increased expression of IFNγ by a variety of T cell lineages, including Tregs. To evaluate the requirement for IFNγ in antitumor responses driven by FS120m treatment, we treated MC38 tumor–bearing C57BL/6 mice with FS120m or isotype control and anti–IFNγ-blocking antibody (Fig. 4A). Consistent with our previous findings (Fig. 2A), treatment with FS120m resulted in significantly reduced tumor growth. However, anti-IFNγ treatment was able to completely reverse this phenotype, showing that IFNγ signaling is essential for the antitumor efficacy of FS120m (Fig. 4B).

### Treg fragility driven by OX40/CD137 dual agonism is partially dependent upon IFNγ signaling

Because blockade of IFNγ signaling could reverse the antitumor efficacy of OX40/CD137 bispecific agonist treatment, we wished to assess whether IFNγ drives changes to the T cell compartment upon treatment with FS120m. Treatment resulted in increased expression of IFNγ by CD8^+^ T cells, CD4^+^ Tconv cells, and Tregs from lymphoid tissues and tumors (Fig. 4C and D). However, combined treatment with FS120m and anti-IFNγ resulted in reduced expression of IFNγ by Tregs compared with FS120m treatment alone, a phenomenon which was only observed consistently in the Treg lineage. This indicates that IFNγ signaling is required to promote Treg fragility upon FS120m treatment.

**Figure 5:**
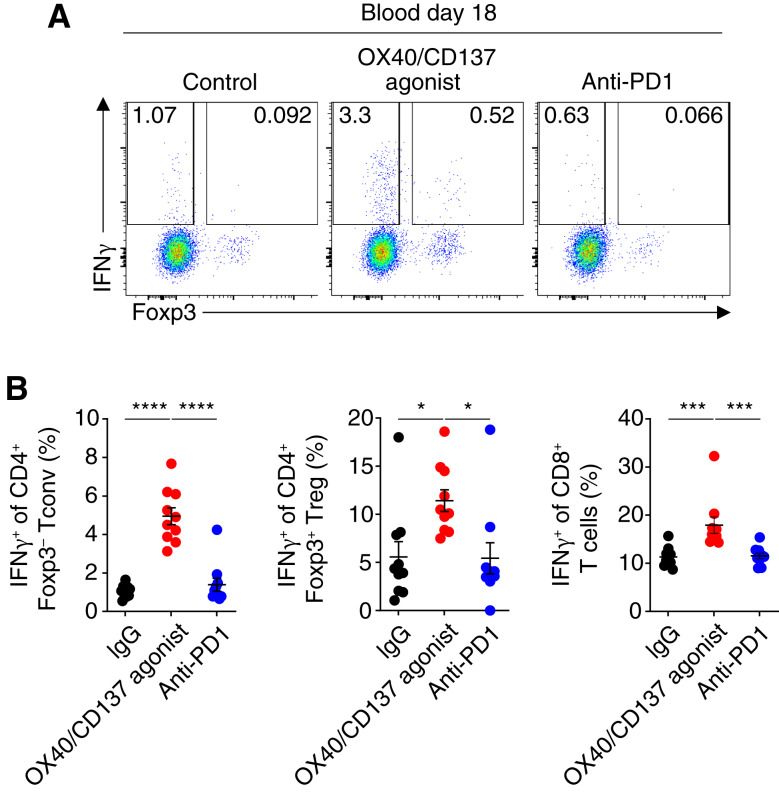
Treg instability is induced by OX40/CD137 bispecific agonist (FS120m) and not anti-PD1 treatment. **A,** Representative plots showing the frequency of IFNγ^+^ cells of CD4^+^ Foxp3^−^ Tconv cells and Foxp3^+^ Tregs in the blood on day 18 after MC38 tumor implantation. **B,** Quantification and statistical analysis of data shown in **A**. Data were analyzed by ordinary one-way ANOVA (**B**) with Tukey correction for multiple comparisons. Bars and error are mean and SEM. *, *P* ≤ 0.05; ***, *P* ≤ 0.001; ****, *P* ≤ 0.0001.

We next asked if induction of Treg fragility is a general property of immunotherapy responses. We compared the ability of FS120m and anti-PD1 therapy to induce IFNγ expression by analyzing the expression of IFNγ in CD4^+^ Tconv and Tregs from the blood of animals treated with FS120m or anti-PD1. We found that FS120m was capable of inducing IFNγ expression in both cell types, whereas anti-PD1 treatment did not share this ability (Fig. 5A and B). This identifies a mechanism of action for OX40/CD137 bispecific agonist treatment that is distinct from that of anti-PD1 and identifies a potential biomarker of FS120m activity.

### IFNγ production by Tregs and/or exTregs is required for full efficacy of OX40/CD137 dual-agonist therapy

**Figure 6:**
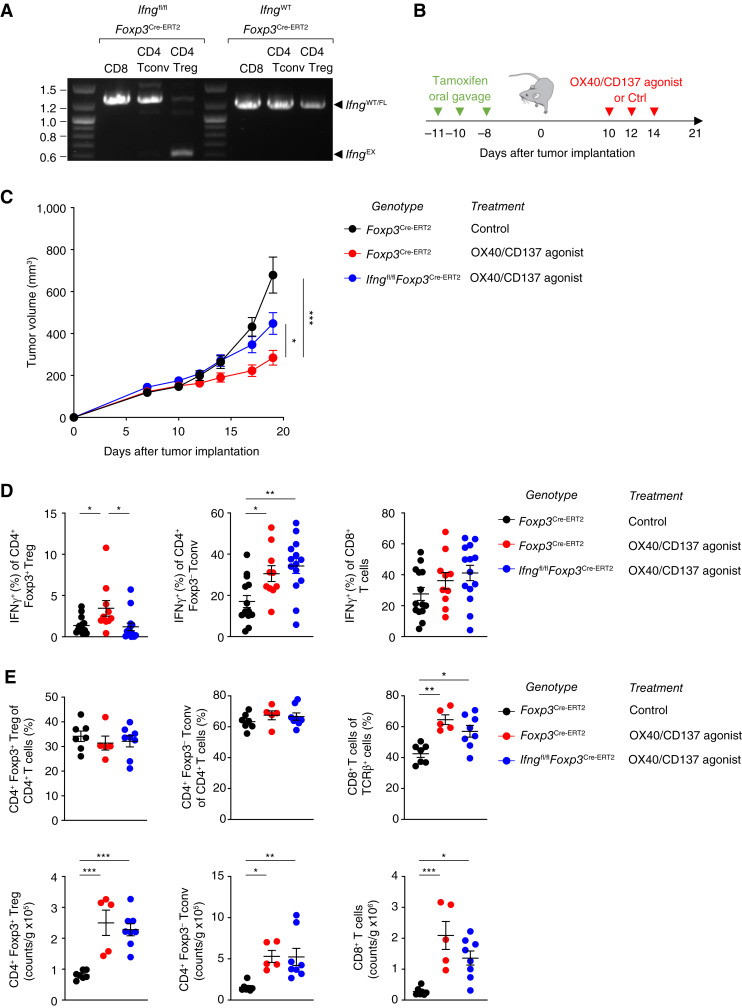
The antitumor efficacy of OX40/CD137 dual agonism is partially dependent upon IFNγ production by Tregs and/or their lineage-instable progeny. **A,** PCR genotyping of CD8^+^, CD4^+^ Foxp3-GFP^−^ Tconv, and CD4^+^ Foxp3-GFP^+^ Tregs sorted from the spleens of mice with the indicated genotypes after treatment with tamoxifen. Tregs have cell-specific excision of IFNγ only in mice possessing both the *Ifng*^fl/fl^ and *Foxp3*^EGFP-Cre-ERT2^ alleles. **B,** Schema representing treatment schedule of tamoxifen and FS120m. **C,** Tumor measurements at indicated timepoints after MC38 implantation of *Ifng*^fl/fl^*Foxp3*^EGFP-Cre-ERT2^ mice and controls given the indicated treatment. **D,** Replicate measurements of IFNγ^+^ cells in the indicated CD4^+^ T-cell populations from the tumor. **E,** Percentages (top) and counts per gram (bottom) of the indicated cell types in the tumors of mice of the indicated genotypes given the specified treatment. Data were analyzed by two-way ANOVA with Tukey correction for multiple comparisons (**C**) and ordinary one-way ANOVA with Tukey correction for multiple comparisons (**D** and **E**). Bars and error are mean and SEM. *, *P* ≤ 0.05; **, *P* ≤ 0.01; ***, *P* ≤ 0.001. Ctrl, control.

Our results showed that Tregs are functionally reprogrammed to produce IFNγ upon OX40/CD137 bispecific agonist treatment, whose efficacy was dependent upon IFNγ production. We therefore wished to test whether the efficacy of FS120m is dependent on IFNγ produced specifically by Tregs and their lineage-instable progeny. To do this, we crossed *Foxp3*^EGFP-Cre-ERT2^ mice with *Ifng*^fl/fl^ mice (47). PCR analysis was used to confirm Treg-specific Cre recombinase activity in this model, given that other constitutive *Foxp3*^Cre^ alleles can induce substantial nonspecific/leaky excision of floxed alleles in non-Treg lineages (56). We treated *Ifng*^fl/fl^*Foxp3*^EGFP-Cre-ERT2^ and *Ifng*^WT^*Foxp3*^EGFP-Cre-ERT2^ animals with tamoxifen and sorted CD8^+^ T cells, CD4^+^ Foxp3-GFP^−^ Tconv cells, and CD4^+^ Foxp3-GFP^+^ Tregs by FACS. Excision of the *Ifng* gene was observed only in Tregs from *Ifng*^fl/fl^*Foxp3*^EGFP-Cre-ERT2^ mice (Fig. 6A), validating use of this system.

To determine whether the efficacy of FS120m is dependent on IFNγ produced specifically by Tregs and their lineage-instable progeny, we treated *Ifng*^fl/fl^*Foxp3*^EGFP-Cre-ERT2^ and *Ifng*^WT^*Foxp3*^EGFP-Cre-ERT2^ mice with tamoxifen before tumor implantation. The mice were then treated with FS120m, and tumor growth was monitored (Fig. 6B). Strikingly, FS120m treatment induced a more effective antitumor response in *Ifng*^WT^*Foxp3*^EGFP-Cre-ERT2^ mice than in *Ifng*^fl/fl^*Foxp3*^EGFP-Cre-ERT2^ mice (Fig. 6C).

The analysis of IFNγ expression by Tregs showed a complete loss of IFNγ^+^ Foxp3^+^ Tregs from *Ifng*^fl/fl^*Foxp3*^EGFP-Cre-ERT2^ mice after treatment with tamoxifen (Fig. 6D). We also found that although treatment with FS120m was associated with an increase in the numbers of infiltrating T cells, deletion of IFNγ within Foxp3^+^ Tregs was associated with a reduction in infiltration by CD8^+^ T cells (Fig. 6E). Because the loss of IFNγ expression was confined to the Foxp3^+^ Treg population, we hypothesize that IFNγ production by fragile and instable Tregs may contribute to increased infiltration of the tumor by CD8^+^ T cells in FS120m-treated mice, resulting in enhanced antitumor immunity. Combined with results indicating that antitumor efficacy is reduced when Tregs are unable to produce IFNγ, we conclude that FS120m exerts its mechanism of action in part by destabilizing Tregs and causing their functional fragility. This reduces their suppressive capability and results in the production of IFNγ which subsequently aids the antitumor response. Together with its direct effects on CD4^+^ Tconv cells and CD8^+^ T cells, this results in enhancement of antitumor immunity.

## Discussion

Overall, our data shows that treatment with the bispecific OX40/CD137 dual-agonist FS120m induces functional reprogramming of murine Tregs, resulting in their production of effector cytokines and loss of their suppressive function, thus promoting tumor regression through both direct and indirect mechanisms.

Tregs are a stable lineage under physiologic conditions; however, it has been previously reported that reduced IL2 signaling can result in lineage instability through the reduction in Foxp3 expression (21). Our data show that FS120m may act through a mechanism that reduces IL2 signaling by reducing CD25 expression on Tregs to induce lineage instability, such that application of exogenous IL2 could restabilize the phenotype. Additionally, it has been shown that exogenous IL2 could reverse an exhausted phenotype induced in Tregs when given anti-OX40 treatment, and this was capable of reinvigorating suppressive capacity in a heart transplant model of long-term allograft survival (38). This highlights the importance of IL2 signaling for the maintenance of Treg function, especially in the presence of costimulatory signaling. Previous preclinical studies have shown that combination therapy using anti-OX40 treatment combined with exogenous IL2 increases antitumor immunity without appreciable effects on Treg suppressive activity (57). While administration of IL2 reduced Treg instability upon FS120 treatment, the late time point of administration of IL2 in our studies precluded our ability to assess the ability of IL2 to potentiate immunotherapy responses driven by FS120 treatment.

Among Tregs which maintain expression of Foxp3, we found that FS120m induces functional fragility. This aligns with previously published findings using therapeutics which target OX40 and CD137 individually. In one such study, *in vivo* anti-OX40 treatment–induced IFNγ and granzyme B expression in Tregs, which was associated with increased T-box expressed in T cells (T-bet) expression (58). In another, anti-CD137 treatment–induced expression of Th-characteristic markers in Tregs that had maintained Foxp3 expression, but which had also begun to express granzyme B, the transcription factor Eomesodermin and TNFα (59). Thus, both molecules targeted by FS120m have the individual capacity to induce some level of Treg fragility. However, when treatment with agonist antibodies targeting OX40 or CD137 individually were compared with FS120m in a CT26 tumor model, it was found that only FS120m was capable of inducing profound antitumor activity (45), indicating that it is only through targeting both pathways simultaneously that tumor regression can be achieved.

The proinflammatory cytokine IFNγ has previously been shown to drive Treg fragility (27). Consistent with these observations, we found that blockade of IFNγ signaling suppressed the increase in IFNγ production induced in Tregs by FS120m treatment. When the *Ifng* gene was specifically disrupted within Tregs, FS120m treatment efficacy was markedly reduced. Importantly, current approaches do not provide the ability to specifically dissect the functional contribution of IFNγ produced by fragile Foxp3^+^ Tregs and their lineage-instable Foxp3^−^ exTreg progeny. However, we found that the absolute number of IFNγ-producing Tregs was greater than that of lineage-instable exTregs upon FS120m treatment, suggesting that fragile Tregs make a more significant contribution to the therapeutic efficacy of OX40/CD137 bispecific agonism.

A key area for future study would be to ask why IFNγ produced by Tregs is so important in driving the therapeutic efficacy of FS120m, given that other Teff-cell subsets (e.g., CD8^+^ T cells) produced more IFNγ upon treatment. It is possible that Tregs and Tconv cells occupy distinct anatomic or microanatomic compartments where the production of IFNγ may yield different and more potent effects. It is relevant in this context to note that Treg fragility and instability were more pronounced in systemic lymphoid tissues than in tumors upon FS120m treatment. It may be that production of IFNγ by Tregs in the systemic lymphoid compartment, tumor-draining lymph nodes, or tertiary lymphoid organs triggers more potent effects on antitumor immunity than production of IFNγ by Tregs in the tumor itself, promoting FS120m therapeutic efficacy. Indeed, experimental ablation of IFNγ production by Tregs was associated with reduced treatment-induced accumulation of CD8^+^ T cells within tumors, which could indicate not only that Treg fragility is required for FS120m to exert its antitumor effects but also that it does so through providing proinflammatory signals that enhance tumor infiltration by cytotoxic CD8^+^ T cells, themselves also shown to have enhanced cytokine production during FS120m treatment. Whether this reflects increased priming of CD8^+^ T cells or improved migration or expansion of CD8^+^ T cells within tumors is unclear. It is plausible that this signal is provided by Tregs within the lymphoid compartment.

In this study, we find that the efficacy of OX40/CD137 dual agonism is in large part dependent upon IFNγ production by Tregs induced upon FS120m treatment. Anti-PD1 therapy did not induce such Treg fragility, revealing a distinct mode of action and supporting combination therapy approaches that exploit this to improve patient outcomes. Indeed, FS120 is currently being evaluated alone, or in combination with anti-PD1 antibodies (pembrolizumab) in a phase I first-in-human study in patients with advanced malignancies (NCT04648202).

## Supplementary Material

Supplementary Figure 1 Supplementary Figure 1: OX40 and CD137 are highly expressed on Treg cells. Representative plots showing OX40+ (left) and CD137+ (right) SP resting or activated cells from the spleen and tumor on day 18 post tumor implantation. ** P ≤ 0.01, *** P ≤ 0.001, **** P ≤ 0.0001. One-way ANOVA with Tukey’s correction for multiple comparisons. Bars and error are mean and s.e.m.

Supplementary Figure 2 Supplementary Figure 2: T cell populations in the spleen and tumor of FS120m treated mice. (A) Percentages of indicated T cell populations in the tumors of mice treated with FS120m or control antibodies. (B) Percentages of indicated T cell populations in the spleens of mice treated with FS120m or control antibodies. (C) Absolute counts of exTreg cells and Foxp3+ RFP+ Treg cells per gram of tumors (left) and in whole spleens (right) of FS120m treated animals. (D) Absolute counts of IFN-γ producing exTreg cells and Foxp3+ RFP+ Treg cells per gram of tumors (left) and in whole spleens (right) of FS120m-treated animals. (E) Absolute counts of TNF producing exTreg cells and Foxp3+ RFP+ Treg cells per gram of tumors (left) and in whole spleens (right) of FS120m-treated animals. * P ≤ 0.05, ** P ≤ 0.01. Student’s t test. Bars and error are mean and s.e.m.

Supplementary Figure 3 Supplementary Figure 3: Increased IL-2 expression by conventional CD4+ and CD8+ T cells with FS120m treatment. Representative plots (left) and replicate measurements (right) of IL-2+ CD4+ Tconv cells and CD8+ T cells in the spleen and draining lymph nodes on day 18 post tumor implantation. *** P ≤ 0.001, **** P ≤ 0.0001. Unpaired Student’s t test. Bars and error are mean and s.e.m.
