# Supplementary material for: IFNγ Production by Functionally Reprogrammed Tregs Promotes Antitumor Efficacy of OX40/CD137 Bispecific Agonist Therapy
Source: Cancer Res Commun. 2024 Aug 12;4(8):2045–57. doi: 10.1158/2767-9764.CRC-23-0500 (PMC11317917; doi:10.1158/2767-9764.CRC-23-0500)
Supplement: Supplementary Figure 1 — OX40 and CD137 are highly expressed on Treg cells. Representative plots showing OX40+ (left) and CD137+ (right) SP resting or activated cells from the spleen and tumor on day 18 post tumor implantation. ** P ≤ 0.01, *** P ≤ 0.001, **** P ≤ 0.0001. One-way ANOVA with Tukey’s correction for multiple comparisons. Bars and error are mean and s.e.m. [file crc-23-0500_supplementary_figure_1_supps1.pptx]

## Slide 1
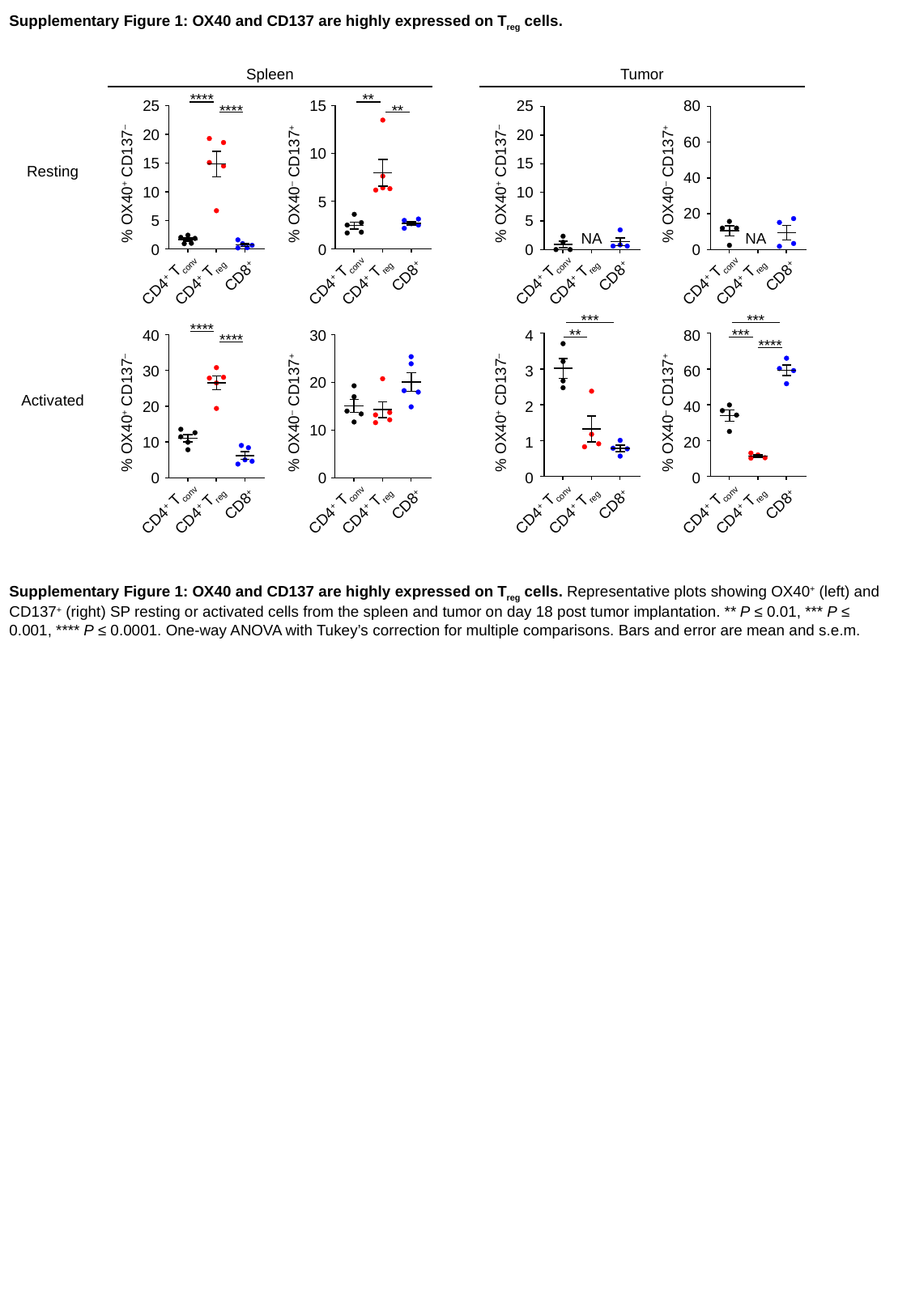

Supplementary Figure 1: OX40 and CD137 are highly expressed on Treg cells.
Spleen
Tumor
****
**
25
80
25
15
****
**
20
20
60
10
15
15
Resting
40
% OX40+ CD137–
% OX40– CD137+
% OX40+ CD137–
% OX40– CD137+
10
10
5
20
5
5
NA
NA
0
0
0
0
CD8+
CD8+
CD8+
CD8+
CD4+ Tconv
CD4+ Tconv
CD4+ Tconv
CD4+ Tconv
CD4+ Treg
CD4+ Treg
CD4+ Treg
CD4+ Treg
***
***
****
**
***
4
80
40
30
****
****
3
60
30
20
Activated
2
40
20
% OX40+ CD137–
% OX40– CD137+
% OX40+ CD137–
% OX40– CD137+
10
1
20
10
0
0
0
0
CD8+
CD8+
CD8+
CD8+
CD4+ Tconv
CD4+ Tconv
CD4+ Tconv
CD4+ Tconv
CD4+ Treg
CD4+ Treg
CD4+ Treg
CD4+ Treg
Supplementary Figure 1: OX40 and CD137 are highly expressed on Treg cells. Representative plots showing OX40+ (left) and CD137+ (right) SP resting or activated cells from the spleen and tumor on day 18 post tumor implantation. ** P ≤ 0.01, *** P ≤ 0.001, **** P ≤ 0.0001. One-way ANOVA with Tukey’s correction for multiple comparisons. Bars and error are mean and s.e.m.
