# Supplementary material for: IFNγ Production by Functionally Reprogrammed Tregs Promotes Antitumor Efficacy of OX40/CD137 Bispecific Agonist Therapy
Source: Cancer Res Commun. 2024 Aug 12;4(8):2045–57. doi: 10.1158/2767-9764.CRC-23-0500 (PMC11317917; doi:10.1158/2767-9764.CRC-23-0500)
Supplement: Supplementary Figure 2 — T cell populations in the spleen and tumor of FS120m treated mice. (A) Percentages of indicated T cell populations in the tumors of mice treated with FS120m or control antibodies. (B) Percentages of indicated T cell populations in the spleens of mice treated with FS120m or control antibodies. (C) Absolute counts of exTreg cells and Foxp3+ RFP+ Treg cells per gram of tumors (left) and in whole spleens (right) of FS120m treated animals. (D) Absolute counts of IFN-γ producing exTreg cells and Foxp3+ RFP+ Treg cells per gram of tumors (left) and in whole spleens (right) of FS120m-treated animals. (E) Absolute counts of TNF producing exTreg cells and Foxp3+ RFP+ Treg cells per gram of tumors (left) and in whole spleens (right) of FS120m-treated animals. * P ≤ 0.05, ** P ≤ 0.01. Student’s t test. Bars and error are mean and s.e.m. [file crc-23-0500_supplementary_figure_2_supps2.pptx]

## Slide 1
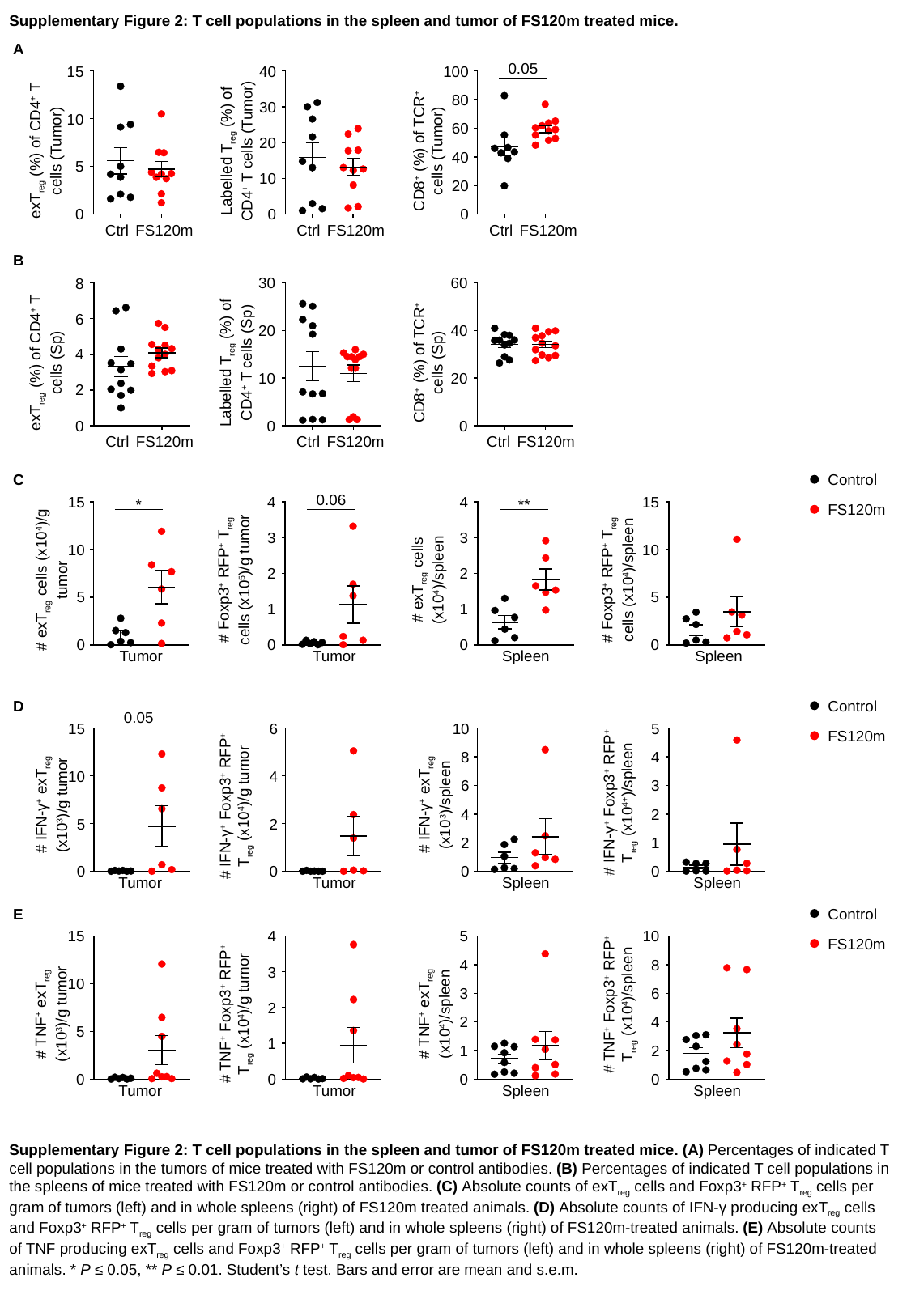

Supplementary Figure 2: T cell populations in the spleen and tumor of FS120m treated mice.
A
15
10
exTreg (%) of CD4+ T cells (Tumor)
5
0
Ctrl
FS120m
40
30
Labelled Treg (%) of CD4+ T cells (Tumor)
20
10
0
Ctrl
FS120m
0.05
100
80
60
CD8+ (%) of TCR+ cells (Tumor)
40
20
0
Ctrl
FS120m
B
8
6
exTreg (%) of CD4+ T cells (Sp)
4
2
0
Ctrl
FS120m
30
20
Labelled Treg (%) of CD4+ T cells (Sp)
10
0
Ctrl
FS120m
60
40
CD8+ (%) of TCR+ cells (Sp)
20
0
Ctrl
FS120m
C
15
*
10
# exTreg cells (x104)/g tumor
5
0
Tumor
0.06
4
3
# Foxp3+ RFP+ Treg cells (x105)/g tumor
2
1
0
Tumor
4
**
3
# exTreg cells (x104)/spleen
2
1
0
Spleen
15
10
# Foxp3+ RFP+ Treg cells (x104)/spleen
5
0
Spleen
Control
FS120m
D
0.05
15
10
# IFN-γ+ exTreg (x103)/g tumor
5
0
Tumor
6
4
# IFN-γ+ Foxp3+ RFP+ Treg (x104)/g tumor
2
0
Tumor
10
8
6
# IFN-γ+ exTreg (x103)/spleen
4
2
0
Spleen
5
4
3
# IFN-γ+ Foxp3+ RFP+ Treg (x104+)/spleen
2
1
0
Spleen
Control
FS120m
E
15
10
# TNF+ exTreg (x103)/g tumor
5
0
Tumor
4
3
# TNF+ Foxp3+ RFP+ Treg (x104)/g tumor
2
1
0
Tumor
5
4
3
# TNF+ exTreg (x104)/spleen
2
1
0
Spleen
10
8
6
# TNF+ Foxp3+ RFP+ Treg (x104)/spleen
4
2
0
Spleen
Control
FS120m
Supplementary Figure 2: T cell populations in the spleen and tumor of FS120m treated mice. (A) Percentages of indicated T cell populations in the tumors of mice treated with FS120m or control antibodies. (B) Percentages of indicated T cell populations in the spleens of mice treated with FS120m or control antibodies. (C) Absolute counts of exTreg cells and Foxp3+ RFP+ Treg cells per gram of tumors (left) and in whole spleens (right) of FS120m treated animals. (D) Absolute counts of IFN-γ producing exTreg cells and Foxp3+ RFP+ Treg cells per gram of tumors (left) and in whole spleens (right) of FS120m-treated animals. (E) Absolute counts of TNF producing exTreg cells and Foxp3+ RFP+ Treg cells per gram of tumors (left) and in whole spleens (right) of FS120m-treated animals. * P ≤ 0.05, ** P ≤ 0.01. Student’s t test. Bars and error are mean and s.e.m.
