# Supplementary material for: IFNγ Production by Functionally Reprogrammed Tregs Promotes Antitumor Efficacy of OX40/CD137 Bispecific Agonist Therapy
Source: Cancer Res Commun. 2024 Aug 12;4(8):2045–57. doi: 10.1158/2767-9764.CRC-23-0500 (PMC11317917; doi:10.1158/2767-9764.CRC-23-0500)
Supplement: Supplementary Figure 3 — Increased IL-2 expression by conventional CD4+ and CD8+ T cells with FS120m treatment. Representative plots (left) and replicate measurements (right) of IL-2+ CD4+ Tconv cells and CD8+ T cells in the spleen and draining lymph nodes on day 18 post tumor implantation. *** P ≤ 0.001, **** P ≤ 0.0001. Unpaired Student’s t test. Bars and error are mean and s.e.m. [file crc-23-0500_supplementary_figure_3_supps3.pptx]

## Slide 1
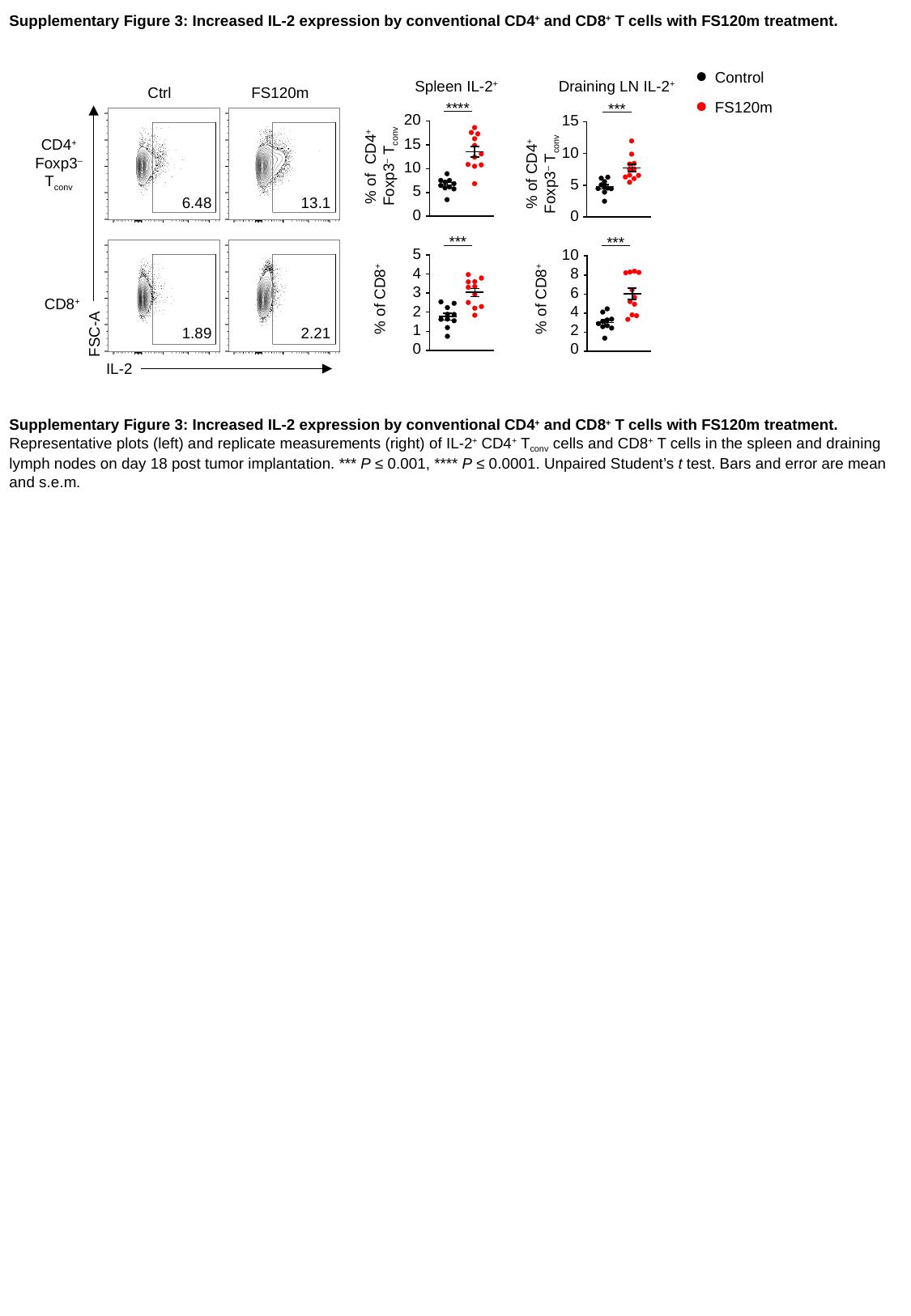

Supplementary Figure 3: Increased IL-2 expression by conventional CD4+ and CD8+ T cells with FS120m treatment.
Control
FS120m
Spleen IL-2+
Draining LN IL-2+
Ctrl
FS120m
****
***
20
15
15
CD4+
Foxp3–Tconv
10
% of CD4+ Foxp3– Tconv
% of CD4+ Foxp3– Tconv
10
5
5
6.48
13.1
0
0
***
***
5
10
4
8
3
6
% of CD8+
% of CD8+
CD8+
2
4
1
2
1.89
2.21
FSC-A
0
0
IL-2
Supplementary Figure 3: Increased IL-2 expression by conventional CD4+ and CD8+ T cells with FS120m treatment.
Representative plots (left) and replicate measurements (right) of IL-2+ CD4+ Tconv cells and CD8+ T cells in the spleen and draining lymph nodes on day 18 post tumor implantation. *** P ≤ 0.001, **** P ≤ 0.0001. Unpaired Student’s t test. Bars and error are mean and s.e.m.
